# A New Two-Photon Ratiometric Fluorescent Probe for Detecting Alkaline Phosphatase in Living Cells

**DOI:** 10.3390/molecules21121619

**Published:** 2016-11-25

**Authors:** Xiaohong Zhou, Yuren Jiang, Xiongjie Zhao, Yao Zhu

**Affiliations:** 1 College of Chemistry and Chemical Engineering, Central South University, Changsha 410083, China; zhouxh001@csu.edu.cn (X.Z.); 152301025@csu.edu.cn (X.Z.); 152311095@csu.edu.cn (Y.Z.); 2 Environment Monitoring Department, Changsha Environmental Protection College, Changsha 410004, China

**Keywords:** two-photon, ratiometric, alkaline phosphatase (ALP), intramolecular charge transfer (ICT)

## Abstract

Alkaline phosphatase (ALP) is an important diagnostic indicator of many human diseases. To quantitatively track ALP in biosystems, herein, for the first time, we report an efficient two-photon ratiometric fluorescent probe, termed probe **1** and based on classic naphthalene derivatives with a donor–π–acceptor (D–π–A) structure and deprotection of the phosphoric acid moiety by ALP. The presence of ALP causes the cleave of the phosphate group from naphthalene derivatives and the phosphate group changes the ability of the intramolecular charge transfer (ICT) and remarkably alters the probe’s photophysical properties, thus an obvious ratiometric signal with an isoemissive point is observed. The fluorescence intensity ratio displayed a linear relationship against the concentration of ALP in the concentration range from 20 to 180 U/L with the limit of detection of 2.3 U/L. Additionally, the probe **1** is further used for fluorescence imaging of ALP in living cells under one-photon excitation (405 nm) or two-photon excitation (720 nm), which showed a high resolution imaging, thus demonstrating its practical application in biological systems.

## 1. Introduction

Alkaline phosphatase (ALP) is a hydrolase that catalyzes the dephosphorylation process of various substrates including nucleic acids, proteins, and carbohydrates [1,2]. As an important diagnostic enzyme, ALP has long been serving as a biomarker in enzyme immunoassays and molecular biology [3], as well as a diagnostic indicator of many human diseases [4]. The normal level of ALP activity in human serum is in a range of 40–150 U/L [5] and the abnormal level of serum ALP are commonly correlated to several diseases such as bone disease, liver dysfunction, breast and prostatic cancer, diabetes and so on [6,7]. Thus, it is highly desirable to develop a sensitive, selective approach to detect ALP in diagnostic and clinical assays.

Among various methods for alkaline phosphatase assay, fluorescence-based assay has drawn much attention because of its simplicity, high sensitivity and real-time detection [8,9,10]. Several fluorescence probes generated from conjugated polyelectrolyte [11,12,13], quantum dots [14,15,16], noble metal nanoclusters [17,18] and organic fluorophores [19,20,21] have been reported for the detection of ALP activities. Despite many achievements from the above-mentioned successful studies, all of them work under one-photon excitation, which requires a rather short excitation wavelength (usually <500 nm). Again, under these conditions, bioimaging applications are affected by such accompanying drawbacks as photobleaching, autofluorescence in cells and tissues, and shallow penetration depth (<100 μm) [22,23,24]. An ideal tool to overcoming such limitations is two-photon microscopy (TPM), which involves fluorophore excitation by two low-energy (longer wavelength) photons [25,26,27,28,29].

**Scheme 1:**
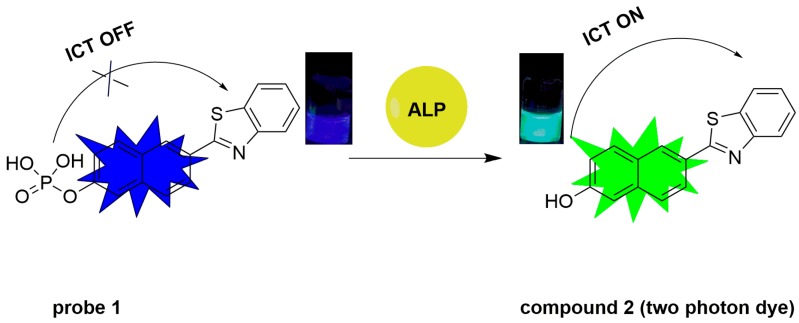
Two-photon ratiometric probe **1**’s structure and detection mechanism toward ALP.

There are many excellent properties, such as large two-photon active absorption cross-section, high fluorescence quantum yield and good photo-chemostability about naphthalene derivatives with a donor–π–acceptor (D–π–A) structure, which has thus been employed extensively as an efficient two-photon platform for designing two-photon probes for various targets [30,31,32,33]. In this work, we have developed a naphthalene-based compound with ICT character as a fluorescent probe (probe **1**, Scheme 1) for the detection of ALP. To the best of our knowledge, until now, there has been no two-photon fluorescent probe for the detection of ALP. As a recognizing group, the phosphate group can be released from the probe **1** in the presence of ALP, and the ICT ability of the phenolic hydroxyl is modulated through dephosphorylation. An ICT process from the cleavage of the phosphate group to the formation of phenolic hydroxyl leads to a bathochromic shift in emission bands, thus affording a two-photon ratiometric fluorescent probe toward ALP [34].

## 2. Results and Discussion

### 2.1. Design and Response Principle of Probe ***1***

**Scheme 2:**
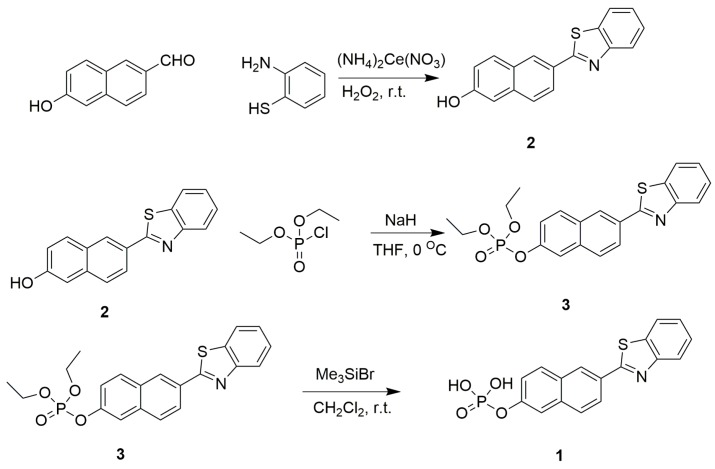
Synthetic route to compound **3** and probe **1**.

**Figure 1:**
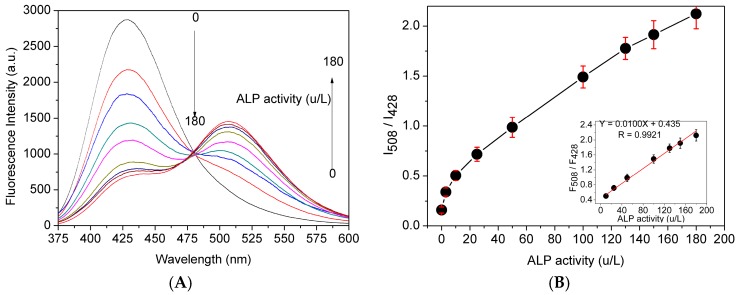
(**A**) Fluorescent spectra of probe **1** (10 μM) in Tris-HCl buffer solution (pH 7.4, 10 mM) with 1% DMSO in the presence of different concentrations of ALP (0–180 U/L) at 37 °C; and (**B**) fluorescent intensity ratio (I_508_/I_428_) as a function of ALP level, insert: the linearity of fluorescent intensity ratio (I_508_/I_428_) as a function of ALP level, λ_Ex_ = 360 nm.

Compound **2** shows excellent optical properties including a large two-photon active absorption cross-section, high fluorescence quantum yield and good photo-chemostability, rendering it a superior candidate for two-photon fluorescent probes [33]. Inspired by the importance of ALP activity assay and typical design strategy of ALP probes, we have modified compound **2** with a phosphate group, which serves as the recognition site of ALP. The phosphate group serving as a weak electron withdrawing group could reduce the electron donating ability of the oxygen atom of phenolic hydroxyl group, thus different ICT intensities occurred before and after the cleavage of phosphate group, resulting in obvious spectral shift and ratiometric signals (Figure S1 and Figure 1A). The sensing mechanism is illustrated in Scheme 1. The synthetic route to probe **1** is depicted in Scheme 2.

### 2.2. Analytical Spectral Properties of Probe ***1***

Concentration-dependent monitoring of enzymatic reaction conducted to examine the possibility of quantitative analysis of ALP activity. As shown in Figure 1A, the fluorescence intensity at 428 nm decreased, while that at 508 nm increased along with ALP activity ranging from 0 to 180 U/L. The fluorescence intensity ratio was plotted versus ALP concentration after probe **1** was incubated with different amount of ALP for 30 min, and they exhibited a good linear relationship through a wide concentration range of 20–180 U/L with the limit of detection of 2.3 U/L (Figure 1B).

**Figure 2:**
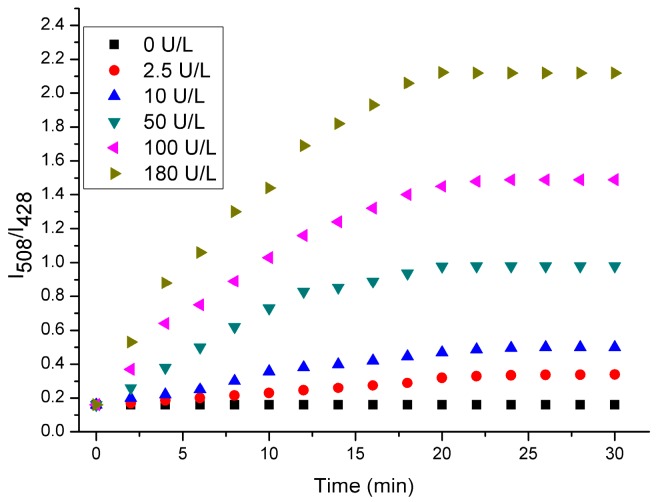
Time-dependent fluorescent spectra of probe **1** (10 μM) in in the presence of different amounts of ALP in Tris-HCl buffer solution (pH 7.4, 10 mM) with 1% DMSO.

We then investigated the time-dependent fluorescent spectra of probe **1** in the presence of ALP at varied concentrations in Tris-HCl buffer solution (pH 7.4, 10 mM) with 1% DMSO. As shown in Figure 2, the fluorescence intensity ratio (I_508_/I_428_) of probe **1** (10 μM) after incubation with different amounts of ALP (0–180 U/L) had different time periods. Obviously, when the concentration of ALP was higher, the fluorescence intensity at 508 nm and the fluorescent intensity ratio (I_508_/I_428_) were enhanced more rapidly. This is understandable by considering the fact that the hydrolysis of the probe would be facilitated in the presence of a high concentration of ALP. Moreover, the fluorescent intensity ratio (I_508_/I_428_) reaches the maximum when the probe is incubated in the presence of ALP for 30 min, indicating that the enzymatic reaction reaches a saturated state.

### 2.3. Effect of pH

The effects of pH on the one-photon fluorescence intensity of 10 μM probe **1** in the absence and presence of 100 U/L ALP were investigated in the pH range from 3 to 10 (Figure S2). As depicted in Figure S2, the emission spectra at 508 nm of probe **1** in the presence of ALP remained stable in the pH range from 6 to 10, and it was decreased in the acidic solution with pH from 5 to 3. This result suggested that probe **1** was able to work over a relatively wide pH range. In this work, we used the physiological pH for the spectral tests.

### 2.4. Selectivity Study

**Figure 3:**
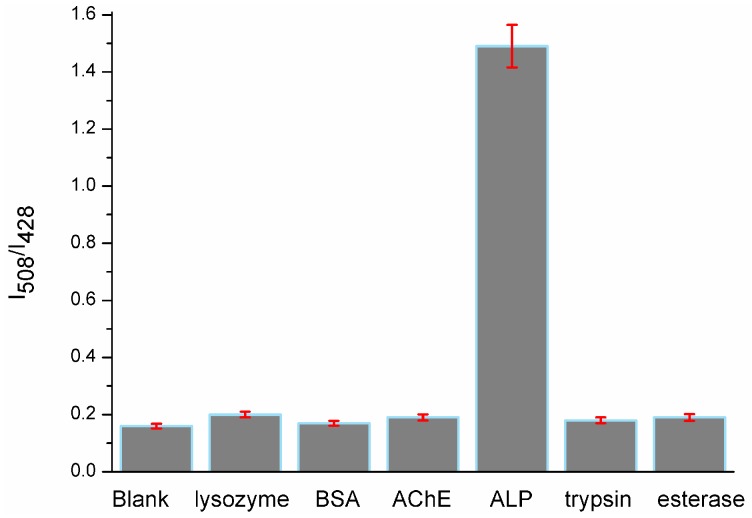
Fluorescent intensity ratio (I_508_/I_428_) changes of probe **1** in the presence of lysozyme, BSA, AChE, ALP, trypsin and esterase in Tris-HCl buffer (pH 7.4, 10 mM) with 1% DMSO.

To examine the selectivity of probe **1** towards phosphatase, we conducted a control experiment by treating probe **1** (10 μM) with other nonspecific proteins, such as lysozyme (100 U/L), bovine serum albumin (BSA, 0.5 mg/mL), acetyl cholinesterase (AChE, 100 U/L), trypsin (100 U/L) and esterase (100 U/L) under the same conditions. The results (Figure 3) showed that no significant fluorescence changes were observed; only the solution mixed with ALP (100 U/L) displayed an intense fluorescence signal at 508 nm. There was about 8.7-fold fluorescent intensity ratio (I_508_/I_428_) enhancement over that of other proteins, which indicated the probe′s high selectivity toward ALP.

### 2.5. TP Fluorescence Propertiess

**Figure 4:**
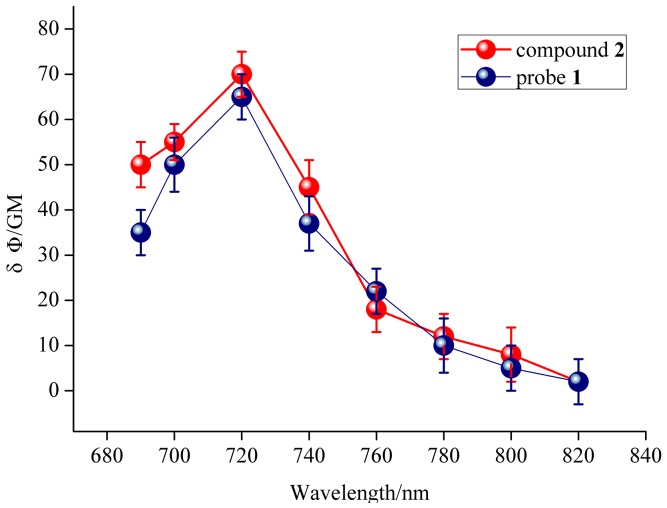
Two-photon action absorption cross-section of probe **1** and compound **2** in CH_3_OH.

The quantum yield for probe **1** was calculated to be 0.41, and the quantum yield for compound **2** was 0.23 in ethanol with rhodamine B as the reference. In order to study the two-photon properties of the probe, the two-photon action absorption cross-section of probe **1** and compound **2** were calculated. Probe **1** was calculated to have 65 GM two-photon action absorption cross-section at 428 nm upon excitation at 720 nm (1 GM = 10^–50^ (cm ^4^ s)/photon) [35] (Figure 4, the blue line), and compound **2** was also calculated with 70 GM at 508 nm upon excitation at 720 nm. These results indicated that the probe **1** and compound **2** have good two-photon action absorption cross-section, and they are potentially useful for bioimaging applications.

### 2.6. Imaging of Endogenous ALP in Living Cells

Inspired by the quantitative analysis of ALP in buffer solution, we further investigated whether the probe was able to monitor endogenous ALP activity in living cells. We first examined cytotoxicity of probe **1** by MTT assay. As shown in Figure S3, there was no significant reduction in cell viability after cells treated with the probe (from 5 μM to 20 μM). The results showed that the probe has low toxicity in vitro.

**Figure 5:**
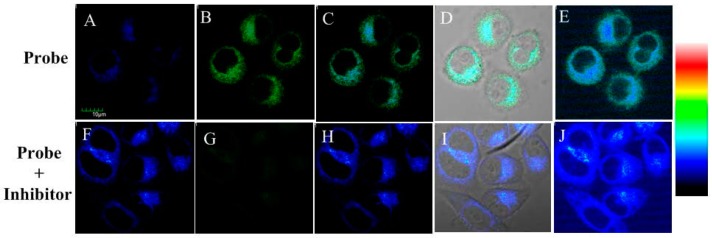
Fluorescence microscopy images for HeLa cells: (**A**–**E**) cells incubated with probe 1 for 30 min; and (**F**–**J**) cells incubated with inhibitor levamisole hydrochloride (5 mM) for 30 min and then with the probe 1 for another 30 min. (**A**,**F**) From blue channel; (**B**,**G**) from green channel; (**C**,**H**) overlay imaging of blue channel and green channel; (**D**,**I**) overlay imaging of blue channel, green channel and bright field; and (**E**,**J**) pseudo color imaging of blue channel and green channel. λ_Ex_ = 405 nm. Blue channel λ_Em_: 410–460 nm and green channel λ_Em_: 470–530 nm. Scale bar: 10 μm.

Then, the probe’s one-photon excited cell imaging was evaluated using Hela cells, in which ALP is overexpressed [36]. The imaging for endogenous ALP in the cells is shown in Figure 5A–E. HeLa cells incubated with the probe showed fluorescence enhancement both in blue and green channel, which is caused by the existence of endogenous ALP in HeLa cells and the production of compound **2**. In the control test, HeLa cells were firstly incubated with ALP inhibitor levamisole hydrochloride for 30 min and then with the probe for another 30 min. As shown in Figure 5F–J, the blue fluorescence was still obvious but the green fluorescence decreased significantly. These results indicated probe **1** could be triggered by endogenic ALP in living cells.

**Figure 6:**
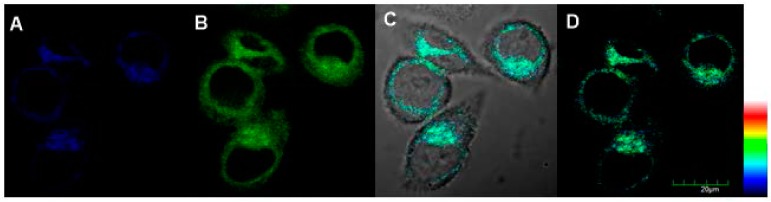
Two-photon fluorescent imaging of HeLa cells treated with probe **1** (10 μM): (**A**) from blue channel; (**B**) from green channel; (**C**) overlay imaging of (**A**,**B**); and (**D**) pseudo color imaging of (**A**,**B**), λ_TPEx_ =720 nm, Blue channel λ_Em_: 410–460 nm and green channel λ_Em_: 470–530 nm, Scale bar: 20 μm.

Two-photon excited confocal fluorescence imaging experiment of probe **1** for endogenic ALP in living cells was then carried out, with results given in Figure 6. The two-photon microscopy imaging showed remarkable two-photon excited fluorescence enhancement after HeLa cells were incubated with 10.0 μM probe **1** for 30 min at 37 °C both in blue channel and green channel (Figure 6). This is because compound **2** has double emission peak, which confirms the hydrolysis and membrane permeability of probe **1**. All of these results also demonstrated that probe **1** was suitable for direct two-photon imaging of ALP in biological samples.

## 3. Experimental Section

### 3.1. Materials and Apparatus

All chemicals were obtained from commercial suppliers and used without further purification. Water was doubly distilled and purified by a Milli-Q system (Millipore, Billerica, MS, USA). NMR spectra were recorded on a Bruker DRX-500 spectrometer (Bruker, Karlsruhe, Germany) using TMS as an internal standard and dimethyl sulfoxide-*d*_6_ (DMSO-*d*_6_) as solvent. Mass spectra were recorded using Agilent 6120 Quadrupole LC/MS from Agilent Technologies (Santa Clara, CA, USA). UV-vis absorption spectra were recorded on a Shimadzu-2450 UV-vis spectrophotometer (Kyoto, Japan). The pH was measured using a pH meter (pHS-3C, Shanghai Leici, Shanghai, China). Fluorescence measurements were carried out on a Hitachi-F4600 fluorescence spectrometer (Hitachi, Kyoto, Japan). Fluorescence images of HeLa cells were obtained using Olympus FV1000-MPE multiphoton laser scanning Confocal microscope (Tokyo, Japan).

### 3.2. Synthesis

Compound **2** was synthesized with 1,2-phenylenediamine and 6-hydroxy-2-naphthaldehyde under mild basic conditions with high yield [37]. Compound **2** was purified by silica gel chromatography with petroleum ether/ethyl acetate (1:1) as eluent to obtain white crystalline products. Compound **3** was then synthesized through phosphorylation of compound **2** with diethyl chlorophosphate in the presence of sodium hydride. Then, compound **3** was deprotected by bromotrimethylsilane to furnish probe **1** [38]. The structures of compound **3** and probe **1** were well characterized using ^1^H-NMR, ^13^C-NMR, ^31^P-NMR and MS (Figures S3–S9).

### 3.3. Optical Property

The fluorescence measurement experiments were measured in buffered (10 mM Tris-HCl, pH 7.4, 2 mM MgCl_2_, 0.2 mM ZnCl_2_) aqueous solvent with 1% DMSO as co-solvent. The pH of buffer solution used was between 3.0 and 10.0. The fluorescent emission spectra were recorded at 375 to 600 nm (the excitation wavelength at 360 nm) and absorption spectra were recorded at 300 to 450 nm. A 1 × 10^−3^ M stock solution of probe **1** was prepared by dissolving probe **1** in DMSO. Procedure of calibration measurements with probe in the buffer was as follows: 10 µL stock solution of probe and 990 µL Tris-HCl buffer solution with different ALP concentration were combined to afford a test solution, which contained 1 × 10^−5^ M of probe **1**.

### 3.4. Cell Viability and Imaging

The cytotoxic effects of the probe **1** were assessed using MTT assays [39]. The living Hela cells were seeded per well in a 96-well plate and incubated for 24 h. Various concentrations (0–20 μM) of the probe **1** were added into the 96-well plate and incubated for an additional 24 h. Then, the cell was treated with MTT (5 mg·mL^−1^). After incubation for 4 h under the same condition, all wells were measured the absorbance at 570 nm, and cell viability of untreated cells was set to 100% as a reference. Each of the experiments was performed at least three times. For fluorescent imaging, cells were cultured in two confocal dishes in the culture medium overnight at 37 °C under a CO_2_ (5%) atmosphere. Then two confocal dishes were treated differently and imaged on an Olympus FV1000-MPE multiphoton laser scanning confocal microscope. In one dish, cells were incubated with probe **1** (10 µM) for 30 min before imaging. In another dish, cells were firstly incubated with ALP inhibitor levamisole hydrochloride (final concentration 5 mM) for 30 min and then with the probe (10 µM) for another 30 min before imaging. One-photon excitation wavelength was fixed at 405 nm, and the two-photon excitation wavelength of the femtosecond laser was fixed at 720 nm. The emission wavelengths were recorded at 410–460 and 470–530 nm respectively.

### 3.5. The Fluorescent Quantum Yields and Two-Photon Excited Fluorescence Measurement

The quantum yields of probe **1** and compound **2** was calculated by comparison with rhodamine B in ethanol as a reference using the following equation:

Φ_F_ = IA_R_(n/n_R)_^2^Φ_R_/I_R_A (1)

where Φ_F_ is the quantum yield, I is the integrated area under the fluorescence spectra, A is the absorbance, n is the refractive index of the solvent, and R refers to the reference rhodamine B.

The two-photon excited fluorescence was measured by using a Ti as described [35]: sapphire femtosecond oscillator (Spectra Physics Mai Tai) as the excitation source. The output laser pulses have a tunable central wavelength from 690 nm to 1020 nm with pulse duration of less than 100 fs and a repetition rate of 80.5 MHz. The laser beam was focused onto the samples using a lens with a focus length of 3.0 cm. The emission was collected at an angle of 90° to the direction of the excitation beam to minimize the scattering. The emission signal was directed into a CCD (Princeton Instruments, Pixis 400B, Acton, MA, USA) coupled monochromator (IsoPlane160) with an optical fiber. A 690 nm short pass filter was placed before the spectrometer to minimize the scattering from the excitation light. The two photon absorption (TPA) cross section (δ) of the sample (s) at each wavelength was calculated according to Equation (2), and rhodamine B in ethanol was used as the reference (r).

Δ = δ_r_ × (S_s_ × Φ_r_ × ϕ_r_ × c_r_)/(S_r_ × Φ_s_ × ϕ_s_ × c_s_) (2)

where S is the integrated fluorescence intensity, Φ is the fluorescence quantum yield, C is the concentration of sample (s) and reference (r), and ϕ is the collection efficiency of the experimental setup. The uncertainty in the measurement of cross sections is ~15%.

## 4. Conclusions

In summary, we have developed the first two-photon ratiometric fluorescent probe **1** for ALP based on the naphthalene derivative. The probe displays desirable properties such as high sensitivity, high selectivity and functioning well at physiological pH. The proposed probe has been used for the determination of ALP in live cells for one- or two-photon fluorescence imaging, which showed high resolution imaging, thus demonstrating its practical application in biological systems.

## Supplementary Materials

Supplementary materials can be accessed at: http://www.mdpi.com/1420-3049/21/12/1619/s1.

## Abbreviations

ALP	alkaline phosphatase
D–π–A	donor–π–acceptor
ICT	intramolecular charge transfer
TPM	two-photon microscopy
I	Intensity
Tris	tris(hydroxymethyl)aminomethane
DMSO	dimethyl sulfoxide
Ex	excitation
BSA	bovine serum albumin
AChE	acetyl cholinesterase
MTT	3-(4,5-dimethyl-2-thiazolyl)-2,5-diphenyltetrazolium bromide
Em	emission
UV	ultraviolet light
LC-MS	liquid chromatography-mass spectrometry
CCD	charge coupled device
Fs	femtosecond
