# Supplementary material for: A New Two-Photon Ratiometric Fluorescent Probe for Detecting Alkaline Phosphatase in Living Cells
Source: Molecules. 2016 Nov 25;21(12):1619. doi: 10.3390/molecules21121619 (PMC6273910; doi:10.3390/molecules21121619)
Supplement: Supplementary file 1 [file molecules-21-01619-s001.pdf]

Supplementary Materials: A New Two-Photon Ratiometric Fluorescent Probe for Detecting Alkaline Phosphatase in Living Cells

Xiaohong Zhou, Yuren Jiang, Xiongjie Zhao and Yao Zhu

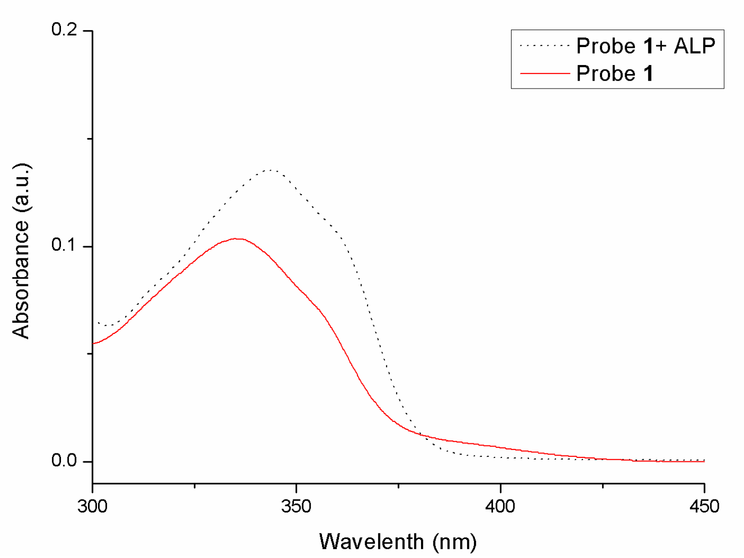

Figure S1. UV-vis absorption spectra of probe **1** (10 μM) and probe **1** (10 μM) + ALP (100 U/L) in Tris-HCl buffer (pH 7.4, 10 mM) with 1% DMSO.

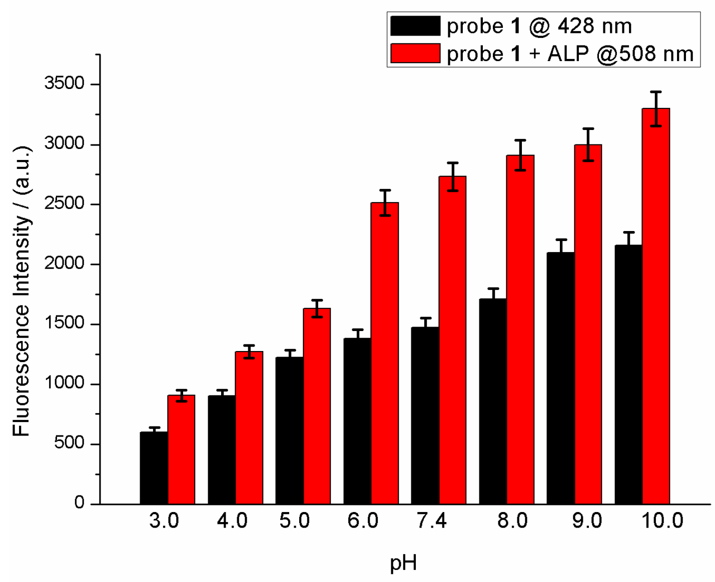

Figure S2. Effect of pH on the peak intensity of probe **1** (10 μM) at 428 nm and the peak intensity of probe **1** (10 μM) in the presence of ALP (100 U/L) at 508 nm in Tris-HCl buffer (pH 7.4, 10 mM) with 1% DMSO.

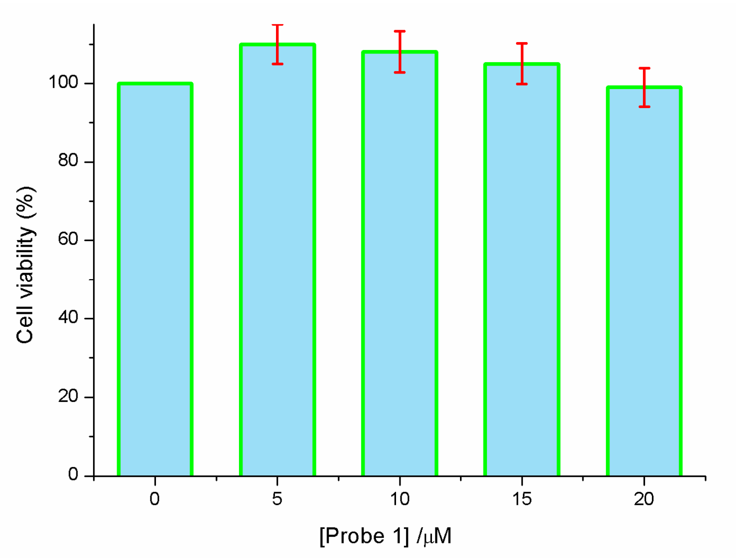

Figure S3. Cytotoxicity of probe **1** (0–20 µM) against HeLa cells, as determined by MTT assay.

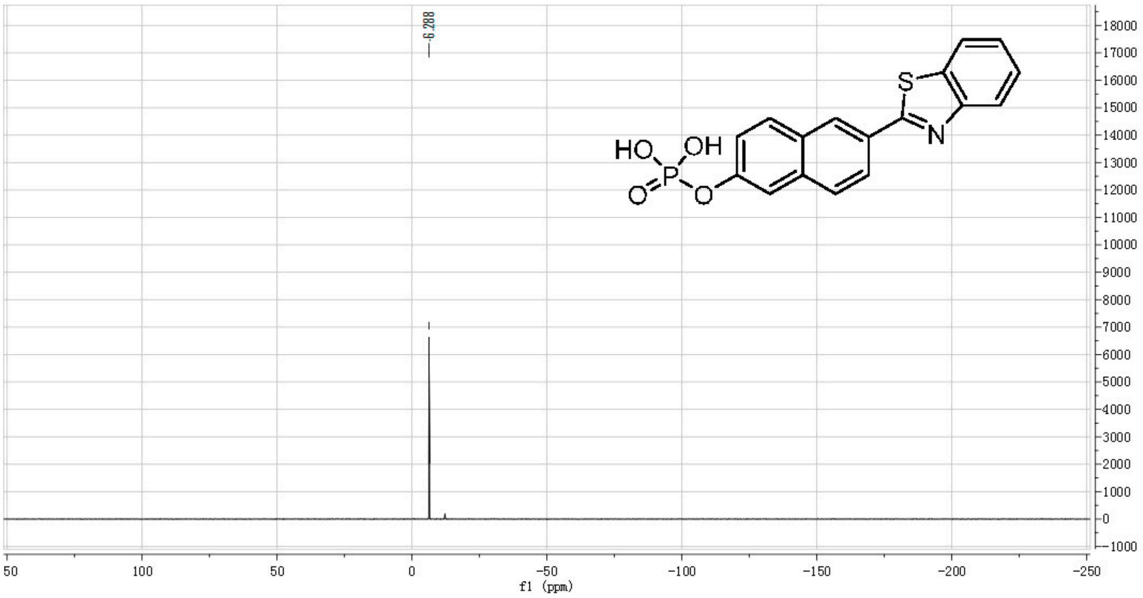

Figure S4. ^{31}P NMR spectra of synthesized probe **1** in DMSO-d_6.

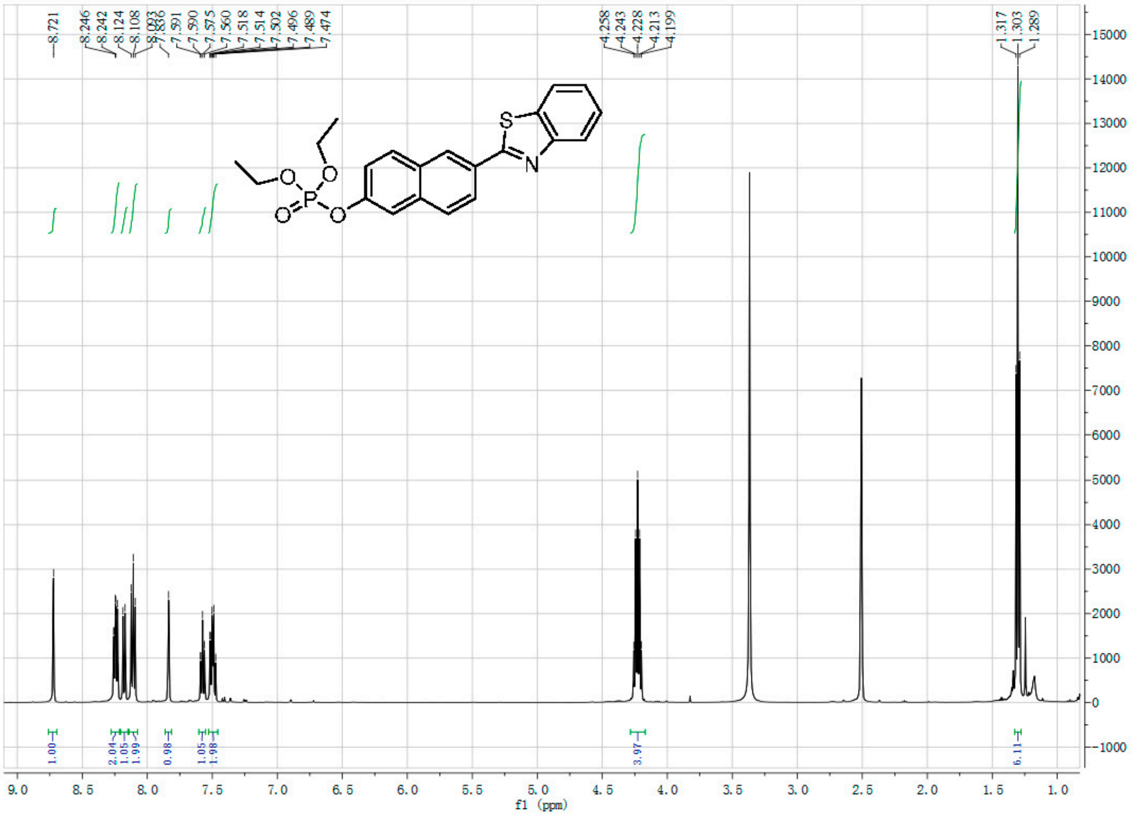

Figure S5. ^{1}H NMR spectra of synthesized compound **3** in DMSO-d_6.

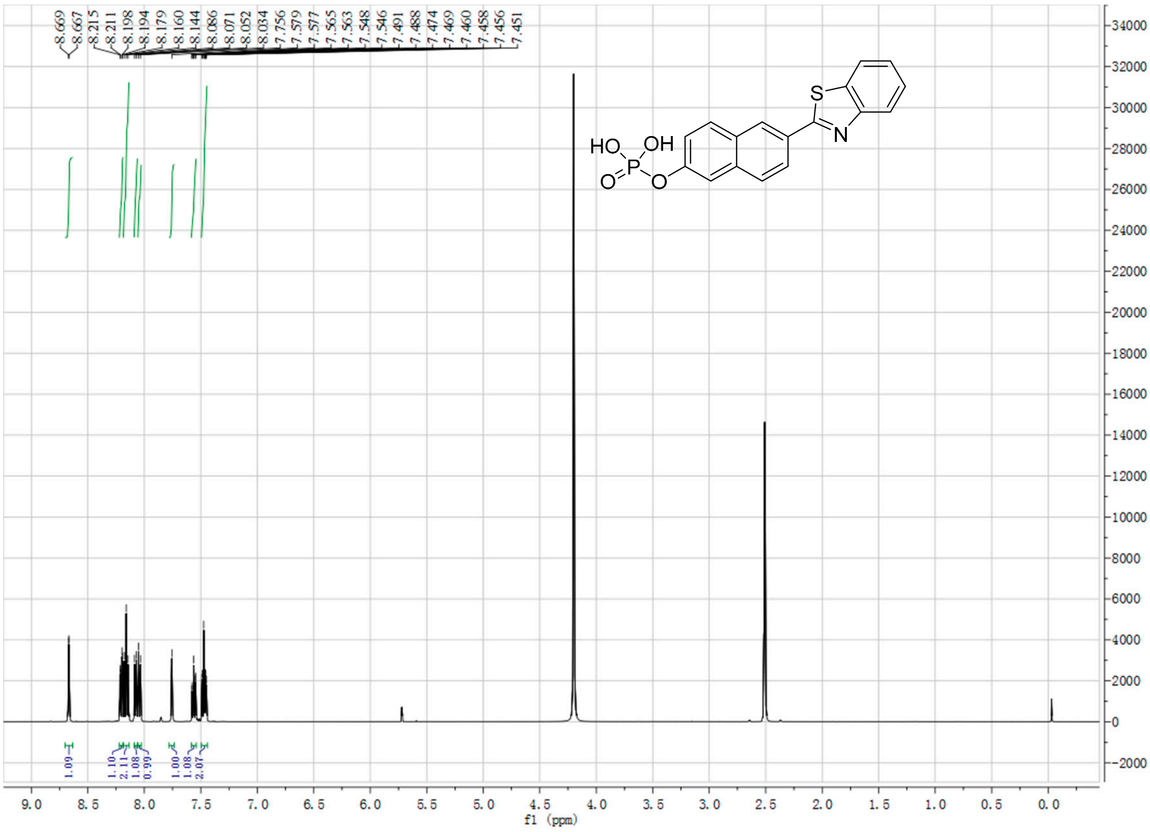

Figure S6. ^{1}H NMR spectra of probe **1** in DMSO-d_6.

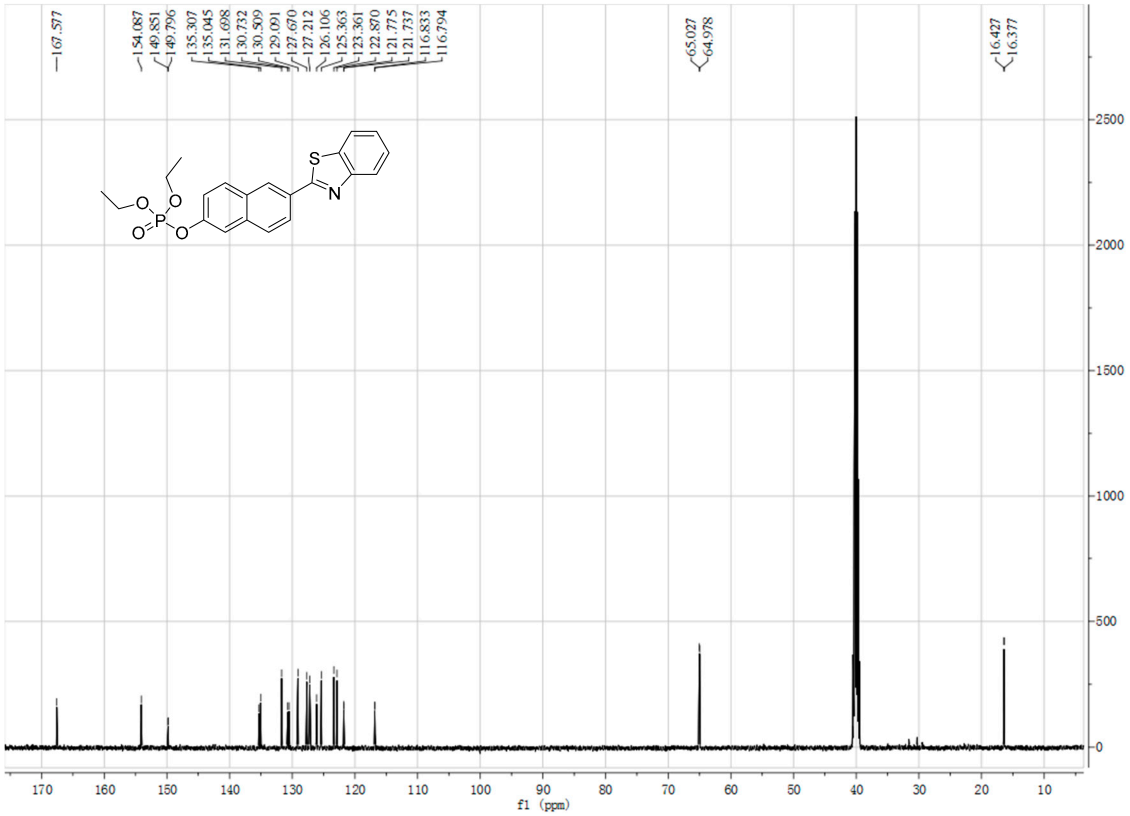

Figure S7. ^{13}C NMR spectra of compound **3** in DMSO-d_6.

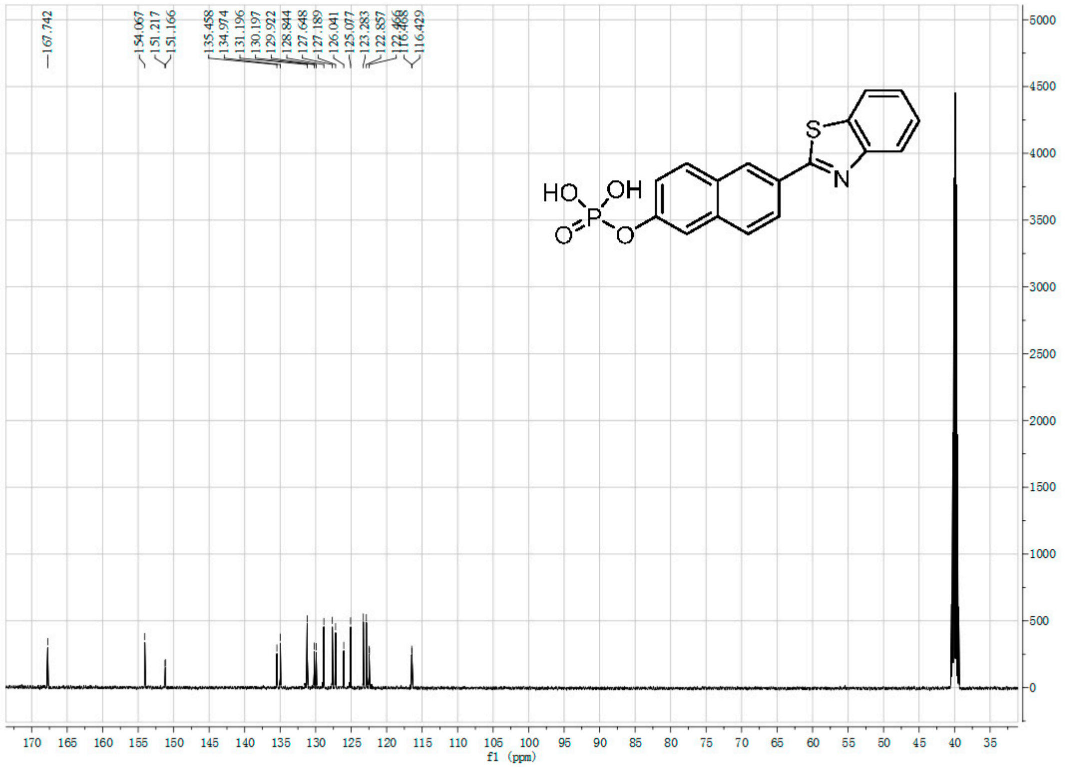

Figure S8. ^{13}C NMR spectra of probe **1** in DMSO-d_6.

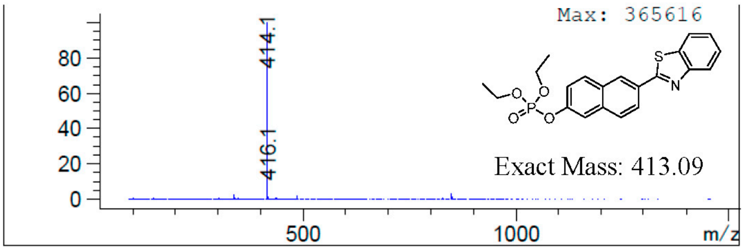

Figure S9. Mass spectrum of compound **3**.

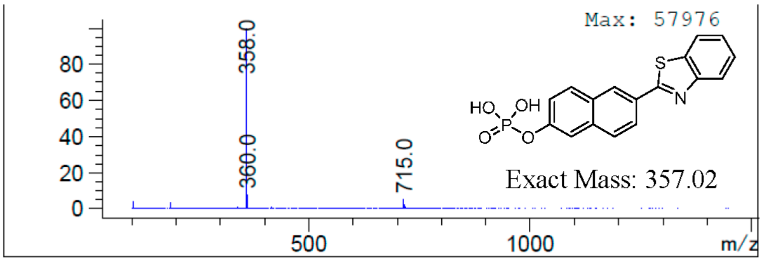

Figure S10. Mass spectrum of probe **1**.

Synthesis of probe **1**:

(1) Synthesis of compound **2**

In a round-bottomed flask (50 mL) equipped with a magnetic stirrer, solutions of 2-aminobenzenethiol (125 mg, 1 mmol) and 6-hydroxy-2-naphthaldehyde (172 mg, 1 mmol) in methyl cyanide (3 mL) were prepared. Hydrogen peroxide (30%, 0.4 mL, 4 mmol,) and ammonium ceric nitrate (54.8 mg, 0.1 mmol) were added and the mixture was stirred at room temperature for 3 h. Then, the reaction mixture was quenched by adding water (10 mL), extracted with ethyl acetate (4 × 10 mL) and dried with anhydrous magnesium sulfate. The filtrate was evaporated and obtained compound **2** (202 mg, yield 89%).

(2) Synthesis of compound **3**

To a solution of **2** (1 mmol, 227 mg) and sodium hydride (2 mmol, 80 mg) in distilled tetrahydrofuran (15 mL), diethyl chlorophosphate (0.3 mL, 2.0 mmol) was added dropwise under nitrogen. The reaction mixture was stirred at room temperature for 3 h. The resulting solution was quenched with water (2 mL), and the solvent was evaporated under reduced pressure. The crude mixture was extracted with ethyl acetate three times. The combined organic layer was dried over magnesium sulfate and filtered, and the solvent was evaporated under reduced pressure. The crude product was purified by silica gel chromatography with petroleum ether/ethyl acetate (5:1) as eluent to afford the desired product (215 mg, yield 52%) as white crystalline products.

^{1}H NMR(500 MHz, DMSO) δ = 8.721 (s, 1H), 8.259–8.229 (m, 2H), 8.187–8.171 (d, 1H), 8.124–8.093 (t, 2H), 7.836 (s, 1H), 7.591–7.560 (dd, 1H), 7.518–7.474 (m, 2H), 4.258–4.199 (m, 4H), 1.317–1.289 (t, 6H); ^{13}C NMR (500 MHz, DMSO) δ = 167.577, 154.087, 149.851–149.796, 135.307, 135.045, 131.698, 130.732, 130.509, 129.091, 127.670, 127.212, 126.106, 125.363, 123.361, 122.870, 121.775–121.737, 116.833–116.794, 65.027–64.978,16.427–16.377; MS (ESI-MS): *m/z* 100% Calcd for $C_{21}H_{21}NO_4PS^+$ [(M + H)$^{1+}$] 414.09; found 414.1.

(3) Synthesis of probe **1**

To a solution of **3** (413 mg, 1 mmol) in dry dichloromethane (25 mL), bromotrimethylsilane (1.4 mL, 10.0 mmol) was added dropwise under nitrogen at 0 °C. The reaction mixture was stirred for 48 h at room temperature. The precipitate was collected by filtration and washed with diethyl ether three times and dried to afford probe **1** as light yellow solid (yield 92%).

^{1}H NMR (500 MHz, DMSO) δ = 8.669–8.667 (d, 1H), 8.215–8.194 (dd, 1H), 8.179–8.144 (t, 2H), 8.086–8.071 (d, 1H), 8.052–8.034 (d, 1H), 7.756 (s, 1H), 7.579–7.546 (m, 1H), 7.491–7.451 (m, 2H); ^{13}C NMR (500 MHz, DMSO) δ = 167.742, 154.067, 151.217–151.166, 135.458, 134.974, 131.196, 130.197, 129.922, 128.844, 127.648, 127.189, 126.041, 125.077, 123.283, 122.857, 122.466, 116.468–116.429; ^{31}P NMR (500 MHz, DMSO) δ = −6.288; MS (ESI-MS): *m*/*z* 100% Calcd for $C_{17}H_{13}NO_4PS^+$ [$(M + H)^{1+}$] 358.02, found 358.0.
